# Editorial Brevities

**Published:** 1887-12

**Authors:** 


### EDITORIALS AND MISCELLANEOUS.

### EDITORIAL BREVITIES.

LAMBERT & CO.—See the advertisement of this excellent firm of St. Louis, to be found on third cover page of this journal.

DR. WETHINGTON, of Thomasville, was recently drowned in a lake where he was fishing. It is supposed he had vertigo which caused him to fall into the lake.

WM. R. WARNER & CO.–This splendid house still continues an interesting advertisement in this journal. We note that a cablegram from London, Oct. 25th, gives them the highest award from American Exhibition in London for superiority of their sugar-coated pills and effervescing salts.

FAIRCHILD BROS. & CO.—We invite attention to the advertisement of this splendid house commencing with to-day's issue. We note that at the American Exhibition, in London, a gold medal was awarded them for "Digestive Ferments," Extractum Pancreatis, Peptonising Powders, Pepsin in Scales; elegant, reliable and convenient preparations for peptonizing food.

A BOOMERANG.—Dr. Kenan, of Baldwin, preferred charges against Dr. Powell, physician in charge of the Lunatic Asylum at Milledgeville, for bad management, waste, maltreatment, etc. The matter was referred to a committee of the Legislature, who not only exculpated Dr. Powell, but severely criticised Dr. Kenan, placing him in a very unenviable attitude before the profession and the public generally.

SURGICAL INSTRUMENTS.—The place to supply yourself with instruments in the surgical line is the establishment of I. Phillips, at 14 Marietta street. Mr. Phillips keeps a good variety, and is a polite, accommodating gentleman. Call and see him. See also his advertisement, on advertising page 20 of this journal.

